# Multiple Dental Anomalies on the Same Side of the Arch in a Single Individual: An Unusual Occurrence

**DOI:** 10.7759/cureus.94095

**Published:** 2025-10-08

**Authors:** Winnifred Christy A, Jones T Raja Devathambi, S Kothai Nachiyar

**Affiliations:** 1 Oral Medicine and Radiology, CSI College of Dental Sciences and Research, Madurai, IND

**Keywords:** distomolar, macrodontia, maxillary first molar, single root, supernumerary tooth

## Abstract

Multiple dental anomalies are often associated with syndromes affecting the orofacial region. Macrodontia refers to the enlargement of a tooth and is relatively rarer than its counterpart, microdontia. Likewise, while the presence of multiple roots and root canals is common in permanent molars, a maxillary molar with a single root and a single root canal is extremely rare. Hyperdontia, or the presence of supernumerary teeth, most commonly occurs in the maxillary anterior and mandibular premolar regions. The occurrence of a maxillary distomolar is uncommon, and the development of a cyst around such a tooth is also rare. This case is reported for its rarity, as it involves multiple anomalies in a single non-syndromic individual, including macrodontia of the maxillary first molar with a single root and root canal, along with the presence of a distomolar on the same side of the maxillary arch.

## Introduction

The term “macrodontia” refers to a morphological anomaly characterized by dental gigantism, which can pose significant clinical challenges. It may result from various factors, including genetic and hormonal influences, or may be associated with syndromes such as insulin-resistant diabetes, otodental syndrome, facial hemihyperplasia, KBG syndrome, Ekman-Westborg-Julin syndrome, and 47,XYY syndrome [1]. Macrodontia can be broadly classified into three types: true generalized (involving all teeth), relative generalized (teeth slightly larger than normal in smaller jaws), and isolated macrodontia (affecting a single tooth) [2].

Supernumerary teeth are additional teeth that develop beyond the normal dental formula, often due to hyperactivity of the dental lamina, atavism, or certain disease processes. They occur in approximately 1-4% of the general population [3]. A supernumerary tooth located distal to the last molar is known as a distomolar or distodens. An accessory fourth molar is also referred to as a distomolar [4].

Developmental anomalies may arise during any stage of tooth development. Root formation results from interactions between the dental epithelium and mesenchymal tissue, and disruption in this process can lead to root anomalies such as dilaceration, concrescence, or accessory roots [5]. The presence of a single root in a maxillary first molar is one such rare occurrence. The simultaneous presentation of macrodontia, a root anomaly, and a supernumerary tooth, along with an early-stage dentigerous cyst (DC) associated with an impacted distomolar on the same side of the maxillary arch, is exceedingly rare. This case report describes such an unusual presentation, emphasizing the clinical features, diagnostic considerations, and management strategies involved.

## Case presentation

A 22-year-old female patient reported to the Department of Oral Medicine and Radiology with a complaint of deposits in the lower front teeth region for the past six months. Her medical, dental, and family history appeared non-contributory. Extraoral examination was normal, and temporomandibular joint examination did not reveal any clicking, popping, or crepitus sounds. Lymph nodes were not palpable, and her face appeared symmetrical.

**Figure 1:**
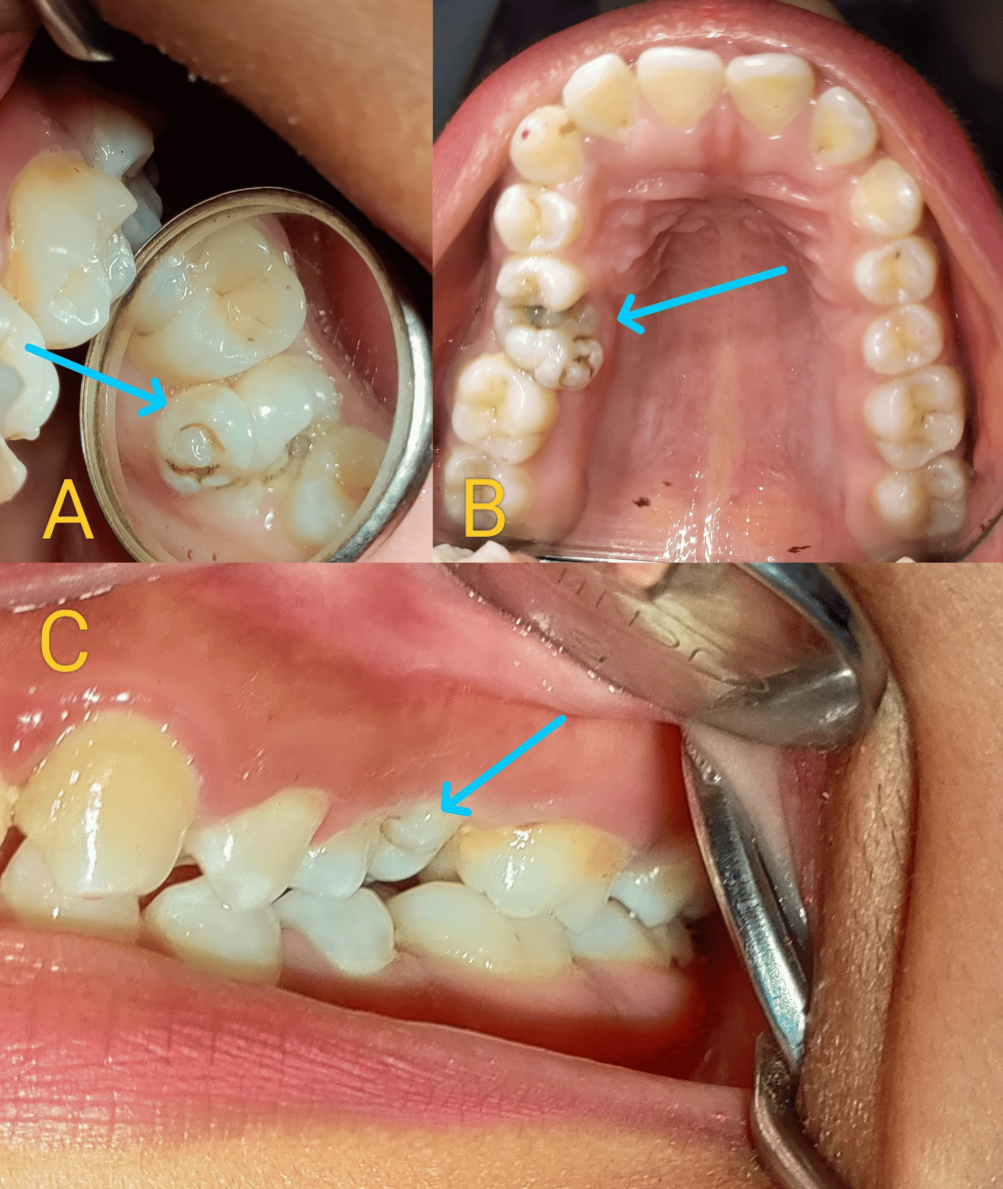
Intraoral photographs (A-C) showing multiple cusps on tooth 26, which is mesially tilted and impinging on the distal surface of tooth 25. Deep caries are evident in teeth 25 and 26.

Intraoral examination revealed the presence of all erupted permanent teeth except for the third molars in the mandible and the right maxillary arch. The maxillary left first molar (26) appeared larger than usual, with approximately eight cusps, and its crown was submerged gingivally on the mesial side (Figure 1A-1C). The patient had no history of pain or sensitivity in the affected tooth.

**Figure 2:**
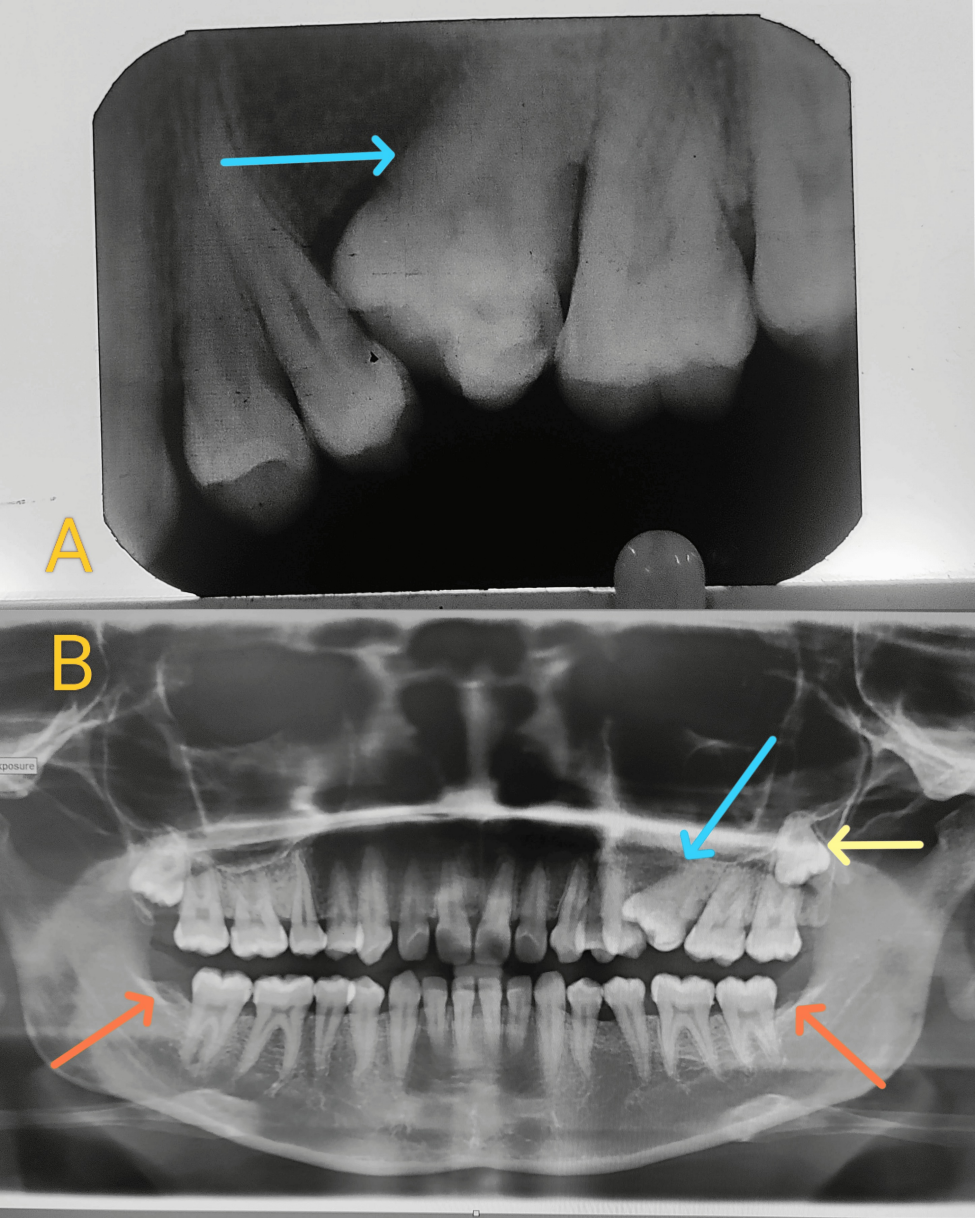
(A) Intraoral periapical radiograph showing a mesially tilted, macrodontic tooth 26 with a single root and single root canal. (B) OPG showing a supernumerary tooth at the apex with pericoronal radiolucency (yellow arrow), a multiple-cusped macrodontic 26 (blue arrow), and congenital absence of the mandibular third molars (orange arrows). OPG, orthopantomogram

The maxillary second premolar (25) had deep caries extending into the pulp space and was tender on percussion. An intraoral periapical radiograph of the tooth revealed unexpected findings. Although an increased number of cusps in cases of macrodontia usually suggests a corresponding increase in the number of roots, it was surprising to find that the macrodontic maxillary first molar, despite its numerous cusps, had only a single root and a solitary canal with a pulp stone (Figure 2A).

The orthopantomogram (OPG) revealed a supernumerary molar impacted distal to the third molar (28), accompanied by a pericoronal radiolucency measuring 1.2 × 1 cm, suggestive of an early-stage DC. Interestingly, the OPG also confirmed the congenital absence of the mandibular third molars (Figure 2B).

**Figure 3:**
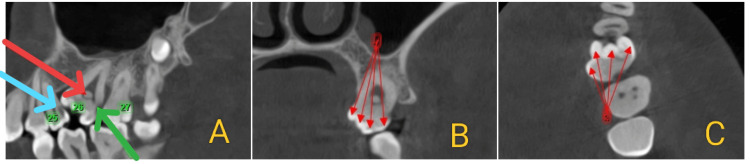
(A) Sagittal section showing a pulpal stone in tooth 26 (red arrow), dental caries involving enamel and dentin in 26 (green arrow), and dental caries involving enamel, dentin, and pulp in 25 (blue arrow). (B, C) Coronal and axial sections showing multiple pulp horns (red arrows) and multiple cusps (red arrows), respectively.

To confirm the presence of a single root and determine the cyst’s position, a cone-beam CT (CBCT) scan was obtained, which showed the following findings: dental caries involving the enamel, dentin, and pulp in 26, and dental caries involving the enamel and dentin in 25. The CBCT also revealed a single root and root canal with a single pulp chamber in 26, which was mesially inclined and impinging on the distal aspect of 25, along with multiple cusps and multiple pulp horns in 26 (Figure 3A-3C).

**Figure 4:**
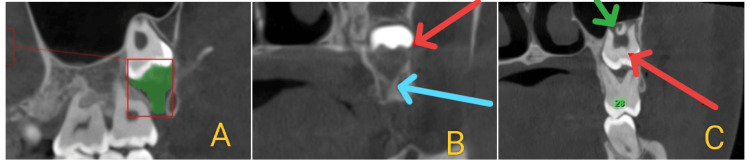
(A) Sagittal section showing a pericoronal radiolucency measuring 1.231 cm³ in volume. (B) Coronal section showing the gubernacular canal apical to tooth 28 (blue arrow) and rupture of the buccal cortex in relation to 28 (red arrow). (C) Coronal section showing an impacted supernumerary tooth (red arrow) and a maxillary sinus lift in relation to 28 (green arrow).

**Figure 5:**
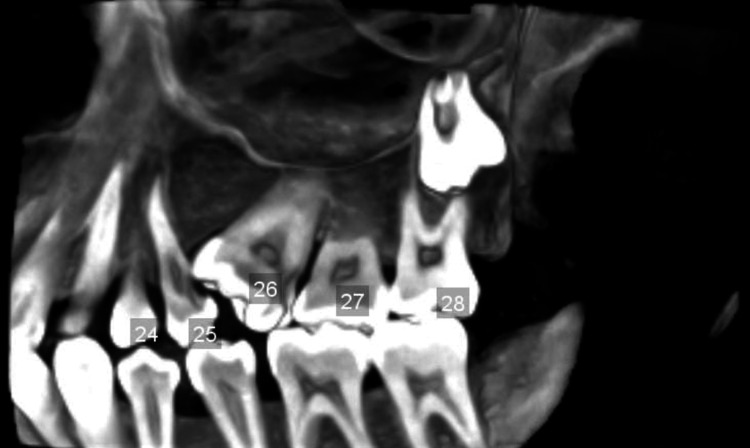
3D reconstructed CT image showing a macrodontic tooth 26 with a pulp stone, single root and root canal, multiple cusps and pulp horns, and an impacted supernumerary tooth apical to 28 with signs of an early DC and congenital absence of the mandibular third molars. DC, dentigerous cyst

The CBCT scan revealed the presence of an impacted supernumerary tooth in the periapical region of 28, associated with a pericoronal radiolucency measuring 9.02 × 8.68 mm in size and a volume of 1.231 cubic centimeters. A “gubernacular canal” was observed in the pericoronal region of the impacted supernumerary tooth, connecting to the alveolar crest. Additionally, rupture of the buccal cortex in relation to 28 was noted, along with elevation of the maxillary sinus floor (Figure 4A-4C). A fully 3D reconstructed image displayed all the findings, including the macrodontic 26 with a pulp stone, single root, and root canal; the impacted supernumerary molar apical to 28 with features of a developing DC; and the congenital absence of the mandibular third molars (Figure 5).

The patient was informed of the proposed treatment plan, which included the extraction of tooth 26, the surgical enucleation of the associated cyst, and the subsequent placement of an implant-supported prosthesis in the 26 region. Potential complications, such as cyst expansion leading to bone destruction and secondary maxillary sinus infection, were also discussed. However, the patient declined immediate intervention due to work constraints. Follow-up evaluations were scheduled on a biannual basis.

## Discussion

Unlike most reported cases of multiple dental anomalies associated with various syndromes, this patient was normal and healthy. The maxillary first molar usually presents with three canals, with variations in the number of root canals ranging from three to seven, as reported by Baratto Filho et al. [6]. A single-rooted maxillary first molar with a single canal is extremely rare and was initially reported by Gopikrishna et al. [7] and Cobankara et al. [8]. To date, fewer than 20 such cases have been reported in the literature. However, none of these cases exhibited an anomalous crown with increased size and multiple pulp horns. Burns described the maxillary first molar as “possibly the most treated, least understood, posterior tooth” [9]. The prevalence of macrodontia in maxillary molars is less than 4%, while the prevalence of a single-rooted maxillary molar varies from 0.25% in the Korean population [10] to less than 0.9% in the Indian population [11]. The presence of both anomalies in a single tooth, as seen in our patient, is considered an exceedingly uncommon occurrence. Interestingly, the presence of pulpal calculi in the crown adds an element of extreme rarity to this case.

Lin et al. reported the highest prevalence of DCs developing in supernumerary teeth as 13.6% [12]. Although distomolars are the most frequently encountered supernumerary teeth after mesiodens, cyst formation from them is infrequent. DCs developing from supernumerary teeth are most commonly found in the anterior maxillary region, followed by the mandibular posterior region. Among the 40 isolated case reports of supernumerary teeth with DCs published to date, none have involved the maxillary posterior region [13]. In our patient, the lesion was located in the maxillary posterior region, which is quite rare. Although DCs arising from supernumerary teeth can occur at any age, they are most frequently observed in the fifth and sixth decades of life, according to published literature. Our patient, being in her early third decade, also represents an uncommon age group for cyst development. To the best of our knowledge, this is the first reported case of this unique combination of anomalies, including an impacted distomolar accompanied by a developing DC on the same side.

## Conclusions

As mentioned earlier, such cases are extremely rare. Most endodontic and dental anatomy texts describe the human maxillary first molar as having three roots and three or four root canals. The present case reports an unusual finding of a macrodontic maxillary first molar with a single root and a single canal containing a pulp stone, with the crown submerged mesially. These variations are associated with an increased risk of dental diseases, and clinicians should be aware of potential complications during treatment to avoid unnecessary risks. Impacted distomolars are usually asymptomatic and are often discovered incidentally during routine radiographic examinations. Although they may appear clinically silent, impacted distomolars can develop into DCs, the second most common odontogenic cyst, which require surgical removal. Careful evaluation of radiographs and internal tooth anatomy is essential for accurate diagnosis and successful treatment.

At present, CBCT is recommended for a more precise diagnosis. CBCT provides 3D information on tooth morphology and positioning and accurately delineates the extent of pathology, enabling more definitive treatment planning. Dental professionals should continually update their knowledge of the various types of dental variations that may occur due to genetic and environmental factors, as it is often said that “the eyes see only what the mind knows.” We present a rare case involving multiple dental anomalies occurring on the same side of the dental arch, a combination not previously reported in the literature.
